# Hypopharyngeal cancer: United Kingdom National Multidisciplinary Guidelines

**DOI:** 10.1017/S0022215116000529

**Published:** 2016-05

**Authors:** P Pracy, S Loughran, J Good, S Parmar, R Goranova

**Affiliations:** 1 Department of ENT/Head and Neck Surgery, Queen Elizabeth Hospital Birmingham, Birmingham, UK; 2 University Department of Otolaryngology, Manchester Royal Infirmary, Manchester, UK; 3 Department of Oncology, University Hospitals Birmingham NHS Foundation Trust, Birmingham, UK; 4 Department of Oral and Maxillofacial Surgery, University Hospitals Birmingham NHS Foundation Trust, Birmingham, UK; 5 Northern Centre for Cancer Care, Newcastle upon Tyne Hospitals, Newcastle upon Tyne, UK

## Abstract

This is the official guideline endorsed by the specialty associations involved in the care of head and neck cancer patients in the UK. With an age standardised incidence rate of 0.63 per 100 000 population, hypopharynx cancers account for a small proportion of the head and neck cancer workload in the UK, and thus suffer from the lack of high level evidence. This paper discusses the evidence base pertaining to the management of hypopharyngeal cancer and provides recommendations on management for this group of patients receiving cancer care.

**Recommendations:**

• Cross-sectional imaging with computed tomography of the head, neck and chest is necessary for all patients; magnetic resonance imaging of the primary site is useful particularly in advanced disease; and computed tomography and positron emission tomography to look for distant disease. (R)

• Careful evaluation of the upper and lower extents of the disease is necessary, which may require contrast swallow or computed tomography and positron emission tomography imaging. (R)

• Formal rigid endoscopic assessment under general anaesthetic should be performed. (R)

• Nutritional status should be proactively managed. (R)

• Full and unbiased discussion of treatment options should take place to allow informed patient choice. (G)

• Early stage disease can be treated equally effectively with surgery or radiotherapy. (R)

• Endoscopic resection can be considered for early well localised lesions. (R)

• Bulky advanced tumours require circumferential or non-circumferential resection with wide margins to account for submucosal spread. (R)

• Offer primary surgical treatment in the setting of a compromised larynx or significant dysphagia. (R)

• Midline lesions require bilateral neck dissections. (R)

• Consider management of silent nodal areas usually not addressed for other primary sites. (G)

• Reconstruction needs to be individualised to the patients’ needs and based on the experience of the unit with different reconstructive techniques. (G)

• Consider tumour bulk reduction with induction chemotherapy prior to definitive radiotherapy. (R)

• Consider intensity modulated radiation therapy where possible to limit the consequences of wide field irradiation to a large volume. (R)

• Use concomitant chemotherapy in patients who are fit enough and consider epidermal growth factor receptor blockers for those who are less fit. (R)

## Introduction

The hypopharynx is subdivided into the piriform sinuses, the posterior pharyngeal wall and the post-cricoid area. The majority of cancers arise in the piriform sinuses (65–85 per cent), 10–20 per cent arise from the posterior pharyngeal wall and 5–15 per cent from the post-cricoid area. As is the case at other sites in the head and neck, the overwhelming majority (95 per cent) of cancers are squamous cell carcinomas (SCCs). Five-year survival is poor with overall survival at 30 per cent, although for T1 and T2 tumours the survival is almost 60 per cent. This discrepancy is a reflection of late presentation, as hypopharynx tumours remain relatively asymptomatic until they are quite advanced. Cases of T1N0 account for only 1–2 per cent of all cases seen and 80 per cent of patients are stage III or IV at presentation. Half of all patients present because of cervical nodes and the incidence of distant metastases at presentation are higher than that for any other head and neck cancer.

## Clinical presentation

The cardinal symptoms of hypopharyngeal cancer are:

• Neck mass, with approximately half of patients presenting such, which reflects the fact that late presentation is common
• Sore throat, particularly if well localised and associated with referred ear pain on swallowing
• Dysphagia, which is progressive and frequently results in significant weight loss and malnutrition
• Hoarseness, voice change and/or upper airway obstruction, a late symptom indicating advanced disease.

## Assessment and staging

### Clinical examination

Assessment of hypopharyngeal cancer requires a full symptomatic history, evaluation of associated medical conditions or comorbidity, determination of weight loss as well as performance status (Karnofsky or World Health Organization). The medical history and performance status are critical in recommending the extent and intention of treatment. Mortality and morbidity rates are much higher in patients with significant weight loss, comorbidity or poor performance indicators.

A full head and neck examination, including nasendoscopy, is necessary in order to assess the size and position of the primary tumour, mobility of the vocal fold and cervical metastases. Clinical examination is also important in assessment of pre-vertebral fascia involvement and can be assessed by examining laryngopharyngeal mobility in the lateral direction. This is then complemented by radiological assessment and staging endoscopy under general anaesthetic.

### Imaging considerations

It is widely agreed that imaging is better performed prior to biopsy, as this can potentially avoid post-operative oedema which may overstage the disease on subsequent imaging. In addition, it allows assessment of any additional abnormalities that have been uncovered by radiological evaluation such as second primary tumours.

Cross-sectional imaging is mandatory in the work up and can take the form of either computed tomography (CT) or magnetic resonance imaging (MRI). In addition to this, the chest should always be imaged due to the increased incidence of lung metastases in advanced hypopharyngeal cancer and to look for synchronous primaries. There is debate about which modality to use. The critical points in imaging are assessing extent of disease (particularly the lower limits of the primary cancer) and the presence of thyroid cartilage invasion. Magnetic resonance imaging gives better soft tissue definition and has greater sensitivity (80 per cent) for cartilage invasion, however, is less specific (74 per cent) than CT, and can therefore potentially overstage disease. The multi-planar capabilities of MR can also help in staging the disease. When compared with histological assessment, CT and MRI produce sensitivities of 66 and 89 per cent, respectively, and specificities of 94 and 84 per cent, respectively. The benefit of CT is that the chest can be imaged at the time of the neck imaging as well as the reduced potential for motion artefact due to the speed of the assessment, whereas, if MRI is used the patient needs additional imaging which may be less convenient for the patient. There is debate whether or not a simple chest X-ray is sufficient or whether CT is necessary. There is evidence to support both arguments, however, as hypopharyngeal cancer usually presents with stage III  or IV  diseases, it seems reasonable to recommend chest CT, as there is a higher incidence of distant metastatic disease in hypopharyngeal cancer.

Currently, the Royal College of Radiologists 2014 guidelines recommends CT or MRI scanning for imaging the hypopharynx.^1^ Computed Tomography should use slice thickness acquired at 0.625–1.25 mm and reformatted at no greater than 2.5 mm for viewing. Scans should be performed during quiet respiration with arms at the side of the patient. Patients should be instructed not to swallow during the evaluation. Magnetic resonance imaging scanning will require a combination of axial, sagittal and coronal T1W and T2W sequences, often with contrast enhancement with spectral fat suppression to assess the extent of soft tissue involvement and cartilage invasion.

Positron emission tomography (PET)–CT is now recommended for assessment of advanced hypopharyngeal primaries, the lower limit of disease in cases not accessible via endoscopy as well as in imaging post-treatment patients to assess for residual and/or recurrent disease.

### Examination under anaesthetic and endoscopy

Endoscopy in theatre serves three functions: first, it allows assessment of the extent of the primary tumour, second, it allows biopsy of the tumour to confirm pathology and finally it allows assessment of other potential primary sites. This last indication was the rationale of the old fashioned triple endoscopy philosophy which incorporated bronchoscopy as well as pharyngolaryngoscopy and oesophagoscopy. It is generally recognised that with the advent of good imaging of the chest the role of formal bronchoscopy has become virtually obsolete.

At the end of all these assessments then a clinical stage can be reached using the tumour–node–metastasis (TNM) classification system (Table I).

**Recommendations**

• Cross-sectional imaging with CT of the head, neck and chest is necessary for all patients; MRI of the primary site is useful particularly in advanced disease; and CT–PET to look for distant disease (R)
• Careful evaluation of the upper and lower extents of the disease is necessary, which may require contrast swallow or CT–PET imaging (R)
• Formal rigid endoscopic assessment under general anaesthetic should be performed (R)

**Table I:** T staging for hypopharyngeal tumours

Tx	Primary tumour cannot be assessed
T0	No evidence of primary tumour
Tis	Carcinoma in situ
T1	Tumour limited to one subsite of the hypopharynx and 2 cm or less in greatest dimension
T2	Tumour invades more than one subsite of the hypopharynx or an adjacent site, or measures more than 2 cm but 4 cm or less in greatest diameter without fixation of hemilarynx
T3	Tumour measures more than 4 cm in greatest dimension or with fixation of hemilarynx
T4a	Tumour invades thyroid/cricoid cartilage, hyoid bone, thyroid gland, oesophagus or central compartment soft tissue, which includes pre-laryngeal strap muscles and subcutaneous fat
T4b	Tumour invades pre-vertebral fascia, encases carotid artery or involves mediastinal structures

## Management

High importance should be placed on exploring patient preferences and involving them in treatment decisions. A clear and unbiased discussion of all options will help the patients and the medical team make the most appropriate decisions. Many of these patients present with dysphagia and significant weight loss and can be profoundly malnourished. This needs to be managed proactively soon after diagnosis and may require insertion of nasogastric or gastrostomy feeding tubes prior to any treatment taking place. A full assessment of the patient's performance status should be carried out to determine their ability to undergo major surgery or their ability to lie flat for radiotherapy and attend daily for seven weeks.

Although some prospective randomised data exists, several aspects of the decision making for hypopharyngeal SCC remain controversial as no treatment has been shown to be superior in terms of disease control and survival.^2^ This section summarises the principles of surgical and non-surgical treatment for these tumours.

**Recommendations**

• Nutritional status should be proactively managed (R)
• Full and unbiased discussion of treatment options should take place to allow informed patient choice (G)

### Surgical treatment

Based on the extent of the tumour, transoral and open surgical options exist for hypopharyngeal cancer.^3^ Transoral approaches have a greater ability to preserve function suitable for smaller tumours where resections can be achieved with clear margins. Radiation therapy is favoured over open partial pharyngeal resections nowadays.

#### Early stage disease

Early stage (I and II) disease can be treated with equal effectiveness with surgery or radiation.^4^^,^^5^ Early lesions of the hypopharynx can be treated by transoral resection or open partial laryngopharyngectomy with or without reconstruction. Surgery offers the advantage of providing prognostic information, such as peri-neural or angioinvasion and lymph node status. This allows the use of post-operative irradiation for those patients likely to gain the most benefit, while sparing other patients side effects without a significant survival advantage. Occult nodal disease is present in 30–40 per cent of patients, so any treatment plan should include elective treatment of the cervical nodes.

#### Late stage disease

Unfortunately, more than 80 per cent are advanced stages III and IV at presentation (with locally advanced disease present in the majority). Submucosal extension is present in more than 60 per cent of surgical specimens and is occult in one-third.^6^ Local recurrence rates have been reported to occur in equal proportion between patients with negative margins and those with positive margins, underscoring the difficulty in clearing disease. Histological studies have reported submucosal extension ranging from 1 to 2 cm, resulting in the recommendation that minimal resection margins of 1.5 cm superiorly, 3 cm inferiorly and 2 cm laterally are required in patients treated surgically. The incidence and extent of submucosal spread is higher in patients who have undergone previous radiotherapy, with macroscopically undetected submucosal spread present in 80 per cent. Bulky advanced tumours will usually require circumferential or non-circumferential resection with free flap cover.

#### Recurrent disease

Surgical salvage after failure of irradiation therapy has a lower success rate for hypopharyngeal cancer than at any other site in the head and neck, and larynx preservation is rarely possible.^7^ Patients who have undergone previous irradiation require even greater resection margins.

**Recommendations**

• Early stage disease can be treated equally effectively with surgery or radiotherapy (R)
• Endoscopic resection can be considered for early well localised lesions (R)
• Bulky advanced tumours require circumferential or non-circumferential resection with wide margins to account for submucosal spread (R)
• Offer primary surgical treatment in the setting of a compromised larynx or significant dysphagia (R)

#### Management of the neck

Midline lesions, those involving the posterior pharyngeal wall or post-cricoid area, and lesions of the medial wall of the piriform sinus, require bilateral neck dissection or irradiation, because of a higher incidence of failure in the contralateral neck. In surgically treated patients with a clinically N0 neck, unilateral or bilateral neck dissection is warranted, depending on the site and size of the primary. In the clinically positive neck, a modified radical neck dissection or a selective neck dissection on one or both sides should be considered. Due attention must be given to nodal involvement of the ‘silent nodal areas’ – retropharynx, parapharynx, paratracheal and mediastinum.

**Recommendations**

• Midline lesions require bilateral neck dissections (R)
• Consider management of silent nodal areas usually not addressed for other primary sites (G)

#### Reconstruction

Reconstruction of pharyngeal defects and in particular circumferential defects present major challenges. Modern chemoradiotherapy protocols, medical comorbidity and poor nutritional status increase surgical morbidity. The aims of reconstruction are to restore swallowing and speech, keeping mortality and morbidity, in particular fistula and stricture rates, to a minimum.

##### Partial pharyngeal defects

Partial pharyngeal defects with more than 3.5 cm of unstretched remaining pharyngeal mucosal width may be closed primarily. Defects with less than 3.5 cm of pharyngeal mucosal width remaining may be reconstructed using a pedicled flap – usually a pectoralis major flap. Free flaps, such as the radial forearm flap and the anterolateral thigh flap may also be used. These reconstructions are also called ‘patch’ grafts. If the pharyngeal mucosal remnant is very narrow (<1 cm in width), some surgeons would recommend excision of the remnant and undertaking a total circumferential reconstruction. However, many surgeons preserve this remnant and reconstruct around it as it may reduce the stricture rate.

##### Total circumferential pharyngolaryngectomy defects

*Lower anastomosis above the clavicles*: Where the lower anastomosis of a total circumferential pharyngolaryngectomy reconstruction would lie above the clavicle, several options exist: jejunal free flap (JFF), gastro-omental free flap (GOFF), tubed radial forearm free flap (RFFF) and a tubed anterolateral thigh free flap (ALT).^8^ All the above options carry the risk of free flap failure, anastomotic leaks, stricturing, donor site morbidity, failure of voice rehabilitation, swallowing problems and a small peri-operative mortality rate.

*Previously untreated cases*: jejunal free flaps have been associated with poorer swallowing thought to be due to uncoordinated peristalsis and wet sounding speech. The RFFF is easy to tube but has donor site issues related to the size of the flap required. Recent literature has suggested that in previously untreated cases, ALTs tubed over a salivary bypass tube appear to provide the lowest complication rates – with minimal donor site morbidity, lower leak rates and lower stenosis rates.^9^ Good swallowing and voice rehabilitation have been reported. However, many authors have not been able to replicate results in the literature and continue to use the JFF. Use of a salivary bypass tube appears to reduce the fistula rates in fasciocutaneous flaps.

*Post-chemoradiotherapy (salvage cases)*: In general, reconstructive surgery using free flap surgery post-chemoradiotherapy carries a higher risk of complications due to the deleterious effects of chemoradiotherapy on tissue vascularity and wound healing. In such cases, limited case series suggest that the use of the GOFF may have an advantage due to the availability and vascularity of the omentum.^10^ The omentum can be wrapped around the anastomotic site to decrease the possibility of leakage and also improve the vascularity of the overlying skin quality. Any of the other options mentioned previously may also be used in the salvage cases. In the patients at high risk of breakdown, a pectoralis major flap may be used to reinforce the anastomotic suture lines in the pharynx.

*Lower anastomosis below the clavicles*: If the resection extends below the clavicles, a gastric pull through or colonic transposition flap may be used.^11^ Both these techniques carry increased morbidity and mortality due to the need to enter multiple visceral cavities. Gastric pull through carries a mortality rate of 5–15 per cent, morbidity of 31–55 per cent and reported fistula rates of 3–23 per cent. Colonic transposition carries similar risks, and appears to be less commonly used. It can however provide a higher cranial reach than gastric pull through, and is therefore useful for tumours that extend up high into the oropharynx.

Swallowing after reconstruction with fasciocutaneous flaps (RFFF and ALT) and GOFF is reported to be superior to that after JFF reconstruction. There is little literature on the outcome of speech rehabilitation following free flap reconstruction of total pharyngeal defects. However, speech rehabilitation is thought to be best when fasciocutaneous flaps are used to reconstruct the pharynx. There is a question as to the advisability of primary tracheoesophageal puncture in these cases. It has been argued that the presence of a puncture site and valve or catheter can increase the chance of infection and flap failure, and for this reason, many surgeons would recommend secondary puncture once the patient has healed and received their post-operative radiotherapy as indicated. Some centres perform a puncture if there is a reasonable distance between the lower anastomosis and the site of the puncture. As there is no evidence to support either position, it is best to decide on an individual case basis and depending on the experience of the team.

**Recommendation**

• Reconstruction needs to be individualised to the patients’ needs and based on the experience of the unit with different reconstructive techniques (G)

### Non-surgical management

Definitive radiotherapy is a potentially organ-sparing alternative to surgery in the treatment of early SCC of the hypopharynx. In combination with systemic therapy, it also has a role in the curative management of locally advanced cancers, although typically not those in which the cartilage is extensively involved or the function of both vocal cords significantly impaired. Post-operative radiation or chemoradiation improves locoregional disease control and overall survival in the presence of well-established high-risk features such as a positive margin or extra-capsular nodal extension of disease.^12^

There has been no randomised side-by-side comparison of surgery and radiotherapy in T1 and 2 N0 hypopharyngeal cancer. In advanced cancers, prospective trials have shown equivalent rates of local control and survival when surgery and adjuvant treatment was compared with primary non-surgical therapy.^13^ Given that the risk of local or locoregional failure is greater than that of distant metastases, cancers that prove radiation resistant are sometimes surgically salvageable. The choice of initial therapy is often driven by pragmatic clinical factors such as age, performance status, medical comorbidity and patient wishes as well as more subjective considerations such as tumour accessibility, local expertise or predicted functional outcome after radiotherapy. A multidisciplinary approach involving surgical and radiation oncologists, speech and language therapists and clinical nurse specialists is required.

The lymphatic drainage of the hypopharynx and the resulting significant risk of occult nodal disease at presentation typically mandate extensive irradiation of at-risk nodal groups as well as treatment of the primary tumour site and clinically apparent nodes. Intensity modulated radiation therapy (IMRT) is now well established in UK radiotherapy centres. This technique, in combination with adherence to consensus guidelines regarding target volume delineation and sophisticated imaging of patient position and anatomical changes during radiotherapy, allows much more precise and accurate targeting of tumouricidal radiation dose to the target.  Intensity modulated radiation therapy also reduces radiation dose to organs at risk, such as the parotid, resulting in reduced medium term toxicity. There is also some evidence that patients treated with IMRT rather than three-dimensional conformal radiotherapy achieve higher rates of local control and better functional outcomes. Intensity modulated radiation therapy should therefore be considered the standard of care.

The predominantly loco-regional pattern of treatment failure in hypopharyngeal cancer has generated interest in treatment intensification, particularly in the setting of locally advanced disease. Intensity modulated radiation therapy has facilitated attempts at escalation of radiation dose. The addition of concomitant systemic therapy in the form of cisplatin (or cetuximab in patients with contraindications such as impaired renal function) confers a modest improvement overall survival at the expense of increased acute toxicity. All but the least fit patients under the age of 71 with stage III or selected stage IV disease should therefore be considered for combination treatment. Patients aged 71 or more were shown in the meta-analysis of chemotherapy in head and neck cancer to be unlikely to benefit from the addition of systemic therapy.^14^^,^^15^

The optimal use of induction chemotherapy in hypopharyngeal cancer, as in other anatomical subsites, remains a topic of discussion. Two large trials have demonstrated its utility in an organ preservation approach with comparable survival to surgery in laryngeal cancer. Induction therapy reduces the incidence of distant metastases but does not have a consistent effect on overall survival, although individual studies comparing induction schedules with and without a taxane have shown a significant benefit for triple-agent chemotherapy.^16^ One pragmatic approach is to offer induction chemotherapy prior to chemoradiation to fit patients with bulky T3 or early T4 disease,^13^ with laryngectomy for those who do not respond to chemotherapy, and to patients at high risk of distant relapse such as those with N2b or c or N3 disease.

**Recommendations**

• Consider tumour bulk reduction with induction chemotherapy prior to definitive radiotherapy (R)
• Consider IMRT where possible to limit the consequences of wide field irradiation to a large volume (R)
• Use concomitant chemotherapy in patients who are fit enough and consider EGFR blockers for those who are less fit (R)

### Palliative care

It has been estimated that up to 25 per cent of patients are not suitable for curative treatment at presentation because of age, the extent of locoregional disease, distant metastases, comorbidity or refusal of surgery. Following treatment, 50–60 per cent of patients develop a recurrence in less than 12 months, and most mortality in the first two years following diagnosis is due to locoregional recurrence. The overall five-year disease specific survival rate is approximately 30–35 per cent with five-year survival rates of 14–22 per cent for stage IV disease. Volume of disease and laryngeal involvement adversely impact survival. Combination chemotherapy has been shown to improve overall survival.^17^

Patients with hypopharyngeal cancer may suffer from severe symptoms; including pain, swallowing difficulties, aspiration, chest infections, anorexia and weight loss. In many cases, symptoms will have been aggravated by previous treatments; surgery, radiation and chemotherapy (mucositis, hypopharyngeal stenosis, infections, pharyngocutaneous fistula, psychological distress and cachexia). All of these require attention and some may be relieved by surgical interventions such as tracheostomy and the insertion of a gastrostomy to relieve breathing and restore hydration and nutrition.

Some patients, with minimal local symptoms are suitable for targeted agents in recurrent local and/or metastatic disease. These are highly selected patients and palliative treatments should be discussed and offered to patients through the multidisciplinary team (MDT). Patients with symptomatic lung metastases are often those who benefit most from palliative chemotherapy. Palliative radiotherapy may be used for patients, unsuitable for curative treatment, who present with bleeding or uncontrolled pain from the hypopharynx and can be excellent for cutaneous metastases, painful lymph nodes or bony disease.

#### Key points

• The majority of cancers arise in the piriform sinuses (65–85 per cent), 10–20 per cent arise from the posterior pharyngeal wall and 5–15 per cent from the post-cricoid area
• Patient choice and involvement in treatment decisions is of high importance and a clear and unbiased discussion of their options will help them and their medical team make the most appropriate treatment decisions
• Primary non-surgical treatment is recommended for most locally advanced tumours unless the laryngeal function is compromised or significant dysphagia exists
• Early stage (I and II) disease can be treated with equal effectiveness with surgery or radiation
• Bulky advanced tumours will usually require circumferential or non-circumferential resection with free flap cover
• Five-year survival is poor with overall survival at 30 per cent, although for T1 and T2 tumours the survival is almost 60 per cent
• Up to 25 per cent of patients are not suitable for curative treatment at presentation because of age, the extent of locoregional disease, distant metastases, comorbidity or refusal of surgery.

## References

[1] Olliff J, Richards P, Connor S, Wong WL, Beale T, Madani G. Head and neck cancers In: Nicholson T, edn. Recommendations for Cross-Sectional Imaging in Cancer Management. London: The Royal College of Radiologists, 2014:3–19

[2] Hall SF, Groome PA, Irish J, O'Sullivan B. Radiotherapy or surgery for head and neck squamous cell cancer: establishing the baseline for hypopharyngeal carcinoma? Cancer 2009;115:5711–22 19708030 10.1002/cncr.24635

[3] Bradley PJ. Cancer of the hypopharynx. Oper Tech Otolaryngol 2005;16:55–66

[4] Eckel HE, Staar S, Volling P, Sittel C, Damm M, Jungehuelsing M. Surgical treatment for hypopharynx carcinoma: feasibility, mortality, and results. Otolaryngol Head Neck Surg 2001;124:561–9 11337663 10.1067/mhn.2001.115060

[5] Martin A, Jackel MC, Christiansen H, Mahmoodzada M, Kron M, Steiner W. Organ preserving transoral laser microsurgery for cancer of the hypopharynx. Laryngoscope 2008;118:398–402 18091337 10.1097/MLG.0b013e31815aeda3

[6] Ho CM, Ng WF, Lam KH, Wei WJ, Yuen AP. Submucosal tumor extension in hypopharyngeal cancer. Arch Otolaryngol Head Neck Surg 1997;123:959–65 9305246 10.1001/archotol.1997.01900090073010

[7] Godballe C, Jorgensen K, Hansen O, Bastholt L. Hypopharyngeal cancer: results of treatment based on radiation therapy and salvage surgery. Laryngoscope 2002;112:834–8 12150614 10.1097/00005537-200205000-00011

[8] Patel RS, Goldstein DP, Brown D, Irish J, Gullane PJ, Gilbert RW. Circumferential pharyngeal reconstruction: history, critical analysis of techniques, and current therapeutic recommendations. Head Neck 2010;32:109–20 19565471 10.1002/hed.21169

[9] Murray DJ, Gilbert RW, Vesely MJ, Novak CB, Zaitlin-Gencher S, Clark JR Functional outcomes and donor site morbidity following circumferential pharyngoesophageal reconstruction using an anterolateral thigh flap and salivary bypass tube. Head Neck 2007;29:147–54 17022086 10.1002/hed.20489

[10] Patel RS, Makitie AA, Goldstein DP, Gullane PJ, Brown D, Irish J Morbidity and functional outcomes following gastro-omental free flap reconstruction of circumferential pharyngeal defects. Head Neck 2009;31:655–63 19260110 10.1002/hed.21016

[11] Triboulet JP, Mariette C, Chevalier D, Amrouni H. Surgical management of carcinoma of the hypopharynx and cervical esophagus: analysis of 209 cases. Arch Surg 2001;136:1164–70 11585510 10.1001/archsurg.136.10.1164

[12] Bernier J, Cooper JS, Pajak TF, van Glabbeke M, Bourhis J, Forastiere A Defining risk levels in locally advanced head and neck cancers: a comparative analysis of concurrent postoperative radiation plus chemotherapy trials of the EORTC (#22931) and RTOG (# 9501). Head Neck 2005;27:843–50 16161069 10.1002/hed.20279

[13] Lefebvre JL, Andry G, Chevalier D, Luboinski B, Collette L, Traissac L Laryngeal preservation with induction chemotherapy for hypopharyngeal squamous cell carcinoma: 10-year results of EORTC trial 24891. Ann Oncol 2012;23:2708–14 22492697 10.1093/annonc/mds065 PMC3457747

[14] Blanchard P, Baujat B, Holostenco V, Bourredjem A, Baey C, Bourhis J Meta-analysis of chemotherapy in head and neck cancer (MACH-NC): a comprehensive analysis by tumour site. Radiother Oncol 2011;100:33–40 21684027 10.1016/j.radonc.2011.05.036

[15] Pignon JP, le Maitre A, Maillard E, Bourhis J, Group M-NC. Meta-analysis of chemotherapy in head and neck cancer (MACH-NC): an update on 93 randomised trials and 17,346 patients. Radiother Oncol 2009;92:4–14 19446902 10.1016/j.radonc.2009.04.014

[16] Posner MR, Hershock DM, Blajman CR, Mickiewicz E, Winquist E, Gorbounova V Cisplatin and fluorouracil alone or with docetaxel in head and neck cancer. N Engl J Med 2007;357:1705–15 17960013 10.1056/NEJMoa070956

[17] Vermorken JB, Mesia R, Rivera F, Remenar E, Kawecki A, Rottey S Platinum-based chemotherapy plus cetuximab in head and neck cancer. N Engl J Med 2008;359:1116–27 18784101 10.1056/NEJMoa0802656

